# Are traditional spinopelvic risk factors relevant for young adults undergoing total hip arthroplasty?

**DOI:** 10.1002/jeo2.70560

**Published:** 2025-11-14

**Authors:** Maxime Rodilla, Luc Lhotellier, Thomas Aubert

**Affiliations:** ^1^ Orthopaedic Department Croix St Simon Hospital Paris France

**Keywords:** hip–spine relationship, impingement, spinopelvic mobility, THA

## Abstract

**Purpose:**

Traditional spinopelvic risk factors, including lumbar stiffness, sagittal imbalance and standing posterior spinopelvic tilt (SPT), are well‐established predictors of impingement in patients undergoing total hip arthroplasty (THA). However, these parameters are related mainly to degenerative lumbar conditions. With the expanding indications for THA in young patients, the relevance of preoperative spinopelvic risk factor assessment and its association with the risks of adverse spinopelvic mobility and impingement in this group remain uncertain.

**Methods:**

We retrospectively analysed a cohort of 730 consecutive patients who underwent THA and had preoperative functional X‐rays and computed tomography scans. The patients were divided into four groups: younger than 50 years, 55 years, 60 years and older than 60 years. We compared the prevalence of spinopelvic risk factors (SPT ≤ −10°, lumbar flexion ≤20°, pelvic incidence–lumbar lordosis mismatch ≥10°), the rate of adverse spinopelvic mobility (∆SPT ≥ 20°) and the in‐silico risk of impingement with a cup orientation of 40° inclination and 20° anteversion. Hip flexion (flexed‐seated pelvic femoral angle [(∆PFA)] was analysed in patients without risk factors across age groups.

**Results:**

Risk factors were absent in patients ≤50 (0%), rare in those ≤55 (4.2%) and ≤60 years (12.0%), but frequent in those >60 years (29.4%). In younger groups, risk factors were not significantly associated with a ΔSPT ≥ 20° or impingement, although both outcomes were observed in 10%–17% of patients. In contrast, in patients >60 years, risk factors were significantly associated with a ΔSPT ≥ 20° (31.6% vs. 15.0%, *p* < 0.001) and impingement (36.7% vs. 27.4%, *p* = 0.039). Across all groups, ΔPFA was greater in patients with adverse spinopelvic mobility, with between‐group differences of +30°–39° in younger patients compared with +17° in older patients.

**Conclusion:**

Traditional risk factor‐based screening may underestimate adverse spinopelvic mobility or impingement risk in younger patients, where excessive hip motion and other factors may be drivers, highlighting the need for functional assessment even in younger patients.

**Level of Evidence:**

Level III, case–control retrospective analysis.

**Abbreviations**
LL: lumbar lordosis
PFA: pelvic femoral angle
PI: pelvic incidence
SPT: spinopelvic tilt
THA: total hip arthroplasty

## INTRODUCTION

While total hip arthroplasty (THA) yields excellent outcomes [22], a small percentage of patients experience complications such as leg length discrepancy [13], residual pain [34], impingement [23] and dislocation [14]. Preoperative planning has become a necessary prerequisite to minimise these risks [11].

The systematic orientation of implants according to Lewinnek's safe zone has limitations, particularly in cases of adverse spinopelvic mobility, where adapting implant positioning could help prevent prosthetic impingement and dislocation [2, 31]. The identification of lumbar pathology, especially sagittal imbalance or lumbar stiffness, allows for the prediction of patients who, when seated, present with excessive anterior pelvic tilt (leading to anterior impingement and posterior dislocation) or, conversely, a posterior pelvic tilt in extension (increasing the risk of posterior impingement and anterior dislocation) [28], with an increased risk of dislocation in the case of standing to flexed seated mobility exceeding 20° (flexed‐seated spinopelvic tilt [ΔSPT] ≥ 20°) [32, 33]. Recent findings also highlight that adverse spinopelvic mobility negatively affects functional recovery and patient‐reported outcomes after THA [26]. In addition, the presence of adverse spinopelvic mobility may lead to impingement when the cup is positioned with a systematic orientation, particularly at 40° of inclination and 15°–20° of anteversion [1, 8]. This requires adapting the cup orientation according to the femoral version and pelvic mobility to minimise this risk. These findings further highlight the importance of identifying at‐risk patients and the potential use of functional planning algorithms [15, 30] or for navigation systems to guide optimal implant positioning [27].

Preoperative risk factors for adverse spinopelvic mobility are now well defined and include lumbar stiffness, posterior pelvic tilt in the standing position and sagittal imbalance assessed through pelvic incidence–lumbar lordosis (PI‐LL) mismatch [21]. However, these factors are associated mainly with degenerative spine disease and increase with age, particularly because of progressive posterior standing SPT and lumbar stiffening over time [20]. However, recent data in hip dysplasia suggest that pelvic tilt may represent an intrinsic morphological feature rather than a compensatory adaptation [16].

The improved longevity of patients who undergo THA [7], especially through innovations and new materials, has led to an expansion of indications for THA in younger patients. Since spinopelvic risk factors increase with age [20], the following question remains: do younger patients also present a risk of adverse spinopelvic mobility or impingement, and are these phenomena linked to classical spinopelvic risk factors? Moreover, hip flexion appears to be an important determinant of pelvic mobility from standing to a flexed seated position [5] and of the risk of impingement. Hip hypermobility may represent a compensatory mechanism for lumbar stiffness, but it can also be constitutional, particularly in ‘hip users’ [28], who typically present with low LL associated with a low PI [4, 6]. It therefore represents an important factor to consider in patients undergoing THA.

The objective of this study was to first analyse spinopelvic risk factors in a continuous cohort of patients who underwent THA, stratified according to age (younger than 50 years, up to 55 years, up to 60 years and older than 60 years), and to assess their association with adverse spinopelvic mobility and the in‐silico risk of prosthetic impingement in a standard cup orientation of 40° inclination and 20° anteversion. Second, the analysis focused on hip flexion from standing to a flexed seated position, according to the presence or absence of adverse spinopelvic mobility.

## METHODS

### Study design and participants

A retrospective series of 730 consecutive patients who underwent primary THA and had available lateral functional radiographs and low‐dose computed tomography (CT) scans taken between January 2020 and May 2025 was included. Preoperative 3D and functional planning using the Optimised Positioning System™ (OPSInsight, Corin, Cirencester, UK) was implemented for patients undergoing cementless THA with ceramic‐on‐ceramic bearings (Meije Dynacup, Corin, Cirencester, UK) or dual‐mobility cups (MobiliT, Corin, Cirencester, UK) [27]. The only exclusion criterion was refusal to participate in the study. The mean age of the patients was 66.52 years (17–93 years), and 459 women (62.9%) and 271 men (37.1%) were included. This study was approved by the local Ethics Committee.

Two lateral X‐rays were taken between 3 months and 6 weeks before surgery for each patient: one of the upper bodies was taken while the patient was standing in a relaxed posture with their feet shoulder width apart, and one was taken while the patient was in a flexed‐seated position, with their femurs parallel to the floor.

### Spinopelvic and pelvic mobility parameters

The measurements taken on the lateral X‐rays included sacral slope, standing and flexed‐seated LL and standing and flexed‐seated SPT measurements. Anterior rotation of the SPT was assigned a positive value, and posterior rotation of the SPT was assigned a negative value. An increase in the SPT denotes an anterior rotation of the pelvis that is equivalent to anteversion, which decreases the PT. PI measurements were made from the bony landmarks on the CT scan. Pelvic mobility during the transition from a standing position to a seated position was measured as the difference between the standing and flexed‐seated SPT (∆SPT). PI–LL mismatch was defined as the difference between PI and LL in the standing position, and lumbar flexion (LF), defined as the difference between standing and flexed‐seated LL.

All the imaging spinopelvic parameters were analysed by two independent engineers [27]. Two surgeons measured the pelvic femoral angle (PFA), defined as the angle that is formed by drawing a line from the centre of the S1 end plate to the centre of the femoral head and drawing a second line parallel to the femoral diaphysis. Hip mobility was measured as the difference between standing and flexed‐seated PFA (∆PFA).

### Outcome

We divided the population into four groups: younger than 50 years, up to 55 years, up to 60 years and older than 60 years.

Classical spinopelvic risk factors were defined according to radiographic thresholds: standing SPT ≤ −10°, LF ≤20° and PI‐LL ≥ 10° [20].

The outcome of interest was adverse spinopelvic mobility, defined as a ΔSPT≥20° between the standing and flexed‐seated positions [32].

For each patient, the risk of prosthetic impingement was simulated in silico with a fixed cup inclination of 40° and an anteversion of 20°. All definitions were established in a radiological and anatomical reference frame using three‐dimensional reconstructions and Murray's conversion algorithms [24]. With respect to head size, we used the head diameter corresponding to the planned cup size (22, 28, 32 or 36 mm). Cup size was planned with reference to the anterior and posterior walls and the superolateral rim on CT scans and was systematically positioned at the acetabular floor. Stem size was selected to achieve optimal metaphyseal and cortical fill on CT planning. Neck length and head size were chosen to restore the preoperative offset. The femur was planned in its anatomical position, that is, with respect to its native anteversion [1, 8].

To specifically assess the role of hip flexion (∆PFA) in the absence of spinopelvic risk factors, analyses were restricted to patients without any risk factors. Patients were stratified by age: ≤50, ≤55, ≤60 and >60 years.

### Statistical analyses

Continuous variables are described as the means and ranges. Normality and heteroskedasticity of data were assessed with the Shapiro–Wilk test and the Levene's test. We compared means and proportions between these groups by using Student's *t‐*tests, analyses of variances (Mann–Whitney tests) or *chi*‐square tests (or Fisher's exact tests if appropriate), and post hoc power was assessed. A value of *p* < 0.05 was considered to indicate statistical significance. All analyses were performed using R (version 4.0.0, R Foundation for Statistical Computing) and EasyMedStat (version 3.27; www.easymedstat.com).

## RESULTS

### Analysis of age in the overall cohort

The characteristics of the population are described in Table 1.

**Table 1:** Baseline characteristics of the patients.

	Patients *n* = 730
Baseline characteristics	
Age, years (SD)	66.5 (11.1)
Sex, female (%)	460 (63)
BMI, kg/m^2^, (SD)	26.3 (4.3)
Spinopelvic parameters	
Standing SPT, ° (SD)	−0.18 (7.8)
Lumbar flexion, °(SD)	48.0 (14.2)
PI‐LL, ° (SD)	−0.86 (12.8)
PI, ° (SD)	55.4 (11.1)
∆PFA, ° (SD)	89.5 (22.5)

Abbreviations: BMI, body mass index; LL, lumbar lordosis; PFA, pelvic femoral angle; PI, pelvic incidence; SD, standard deviation; SPT, spinopelvic tilt.

There were 730 patients. The indications for THA were primary osteoarthritis (94.4%), osteonecrosis of the femoral head (2.9%), hip dysplasia (0.3%), sequelae of slipped capital femoral epiphysis (0.6%), acetabular or femoral fracture sequelae (0.4%), femoral dysplasia (1%), ankylosing spondylitis (0.3%) and osteochondritis (0.1%).

### Spinopelvic risk factors, adverse spinopelvic mobility and risk of impingement

Among patients ≤50 years (*n* = 57), no risk factors were identified, while a ΔSPT ≥ 20° and a risk of impingement were observed in 8.7% and 14.1% of the patients, respectively. In the ≤55‐year‐old group (n = 96), the presence of one or more risk factors was present in only 4.2%, whereas ΔSPT ≥ 20° and the risk of impingement occurred in 9.4% and 16.8% of patients, respectively. In patients ≤60 years (*n* = 192), the presence of risk factors was slightly more frequent (12.0%), while ΔSPT ≥ 20° (10.4%) and impingement (16.2%) remained similar.

**Figure 1:**
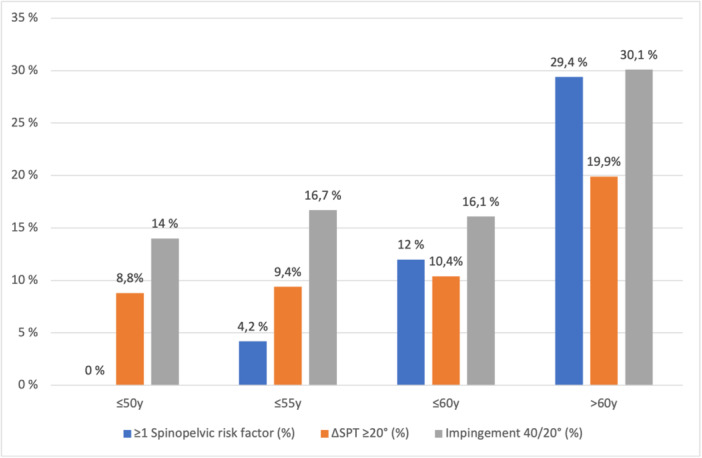
Prevalence of spinopelvic risk factors, adverse spinopelvic mobility (ΔSPT ≥ 20°) and risk of impingement (40/20°) across age groups. SPT, spinopelvic tilt.

In patients older than 60 years, spinopelvic risk factors were frequent, with almost one‐third (29.4%) presenting at least one. Adverse spinopelvic mobility (ΔSPT ≥ 20°) was present in 19.9% and the risk of impingement in 30.1%, both significantly higher than in patients ≤60 years (*p* = 0.004 and *p* < 0.001, respectively; Figure 1 and Table 2).

**Table 2:** Spinopelvic risk factors, adverse spinopelvic mobility and the risk of impingement are associated with patient age.

	Patients ≤50 years of age	Patients >50 years of age	*p* value	Patients ≤55 years of age	Patients >55 years of age	*p* value	Patients ≤60 years of age	Patients >60 years of age	*p* value
	*n* = 57 (7.8%)	*n* = 673 (92,2%)		*n* = 96 (13.1%)	*n* = 634 (86.8%)		*n* = 192 (26.3%)	*n* = 538 (73.7%)	
Spinopelvic risk factors									
Standing SPT ≤ −10°, *n* (%)	0 (0%)	91 (13.5%)	**<0.001**	3 (3.1%)	88 (13.9%)	**0.005**	11 (5.7%)	80 (14.9%)	**0.002**
Lumbar flexion ≤20°, *n* (%)	0 (0%)	136 (20.2%)	**<0.001**	2 (2.1%)	134 (21.2%)	**<0.001**	9 (4.7%)	127 (23.6%)	**<0.001**
PI‐LL ≥ 10°, *n* (%)	0 (0%)	137 (20,4%)	**<0.001**	2 (2.1%)	135 (21,3%)	**<0.001**	16 (8.3%)	121 (22,5%)	**<0.001**
Spinopelvic risk factors ≥1, *n* (%)	0 (0%)	181 (26.9%)	**<0.001**	4 (4.17%)	177 (27.9%)	**<0.001**	23 (12.0%)	158 (29.4%)	**<0.001**
∆SPT ≥ 20°, *n* (%)	5 (8.7%)	122 (18.1%)	0.108	9 (9.4%)	118 (18.6%)	**0.037**	20 (10.4%)	107 (19.9%)	**0.004**
Impingement 40/20°, *n* (%)	8 (14.1%)	185 (27.5%)	0.039	16 (16.8%)	177 (27.92%)	**0.031**	31 (16.2%)	162 (30.1%)	**<0.001**

*Note*: Bold values indicate statistically significant (*p* < 0.05).

Abbreviations: LL, lumbar lordosis; PI, pelvic incidence; SPT, spinopelvic tilt.

### Association between spinopelvic risk factors, adverse spinopelvic mobility and impingement across age groups

In younger patients (≤50, ≤55 and ≤60 years), statistical analyses revealed no significant association between the presence of risk factors and a ΔSPT ≥ 20° or the risk of impingement (all *p* > 0.7). However, both outcomes remained present in 10%–17% of patients without risk factors.

In contrast, in patients >60 years (*n* = 538), the presence of one or more risk factors was frequent (29.4%) and significantly associated with both a ΔSPT ≥ 20° (31.6% vs. 15.0%; *p* < 0.001) and the risk of impingement (36.7% vs. 27.4%; *p* = 0.039; Table 3).

**Table 3:** Associations between spinopelvic risk factors, adverse spinopelvic mobility (ΔSPT ≥ 20°) and the risk of impingement across age groups.

	No spinopelvic risk factor	≥1 spinopelvic risk factor	*p* value
Patient ≤50 years	57 (100%)	0 (0%)	
∆SPT ≥ 20°, *n* (%)	5 (8.7%)	0 (0%)	>0.999
Impingement 40/20°, *n* (%)	8 (14.4%)	0 (0%)	>0.999
Patient ≤55 years	92 (95.8%)	4 (4.2%)	
∆SPT ≥ 20°, *n* (%)	9 (9.8%)	0 (0%)	>0.999
Impingement 40/20°, *n* (%)	16 (17.4%)	0 (0%)	>0.999
Patient ≤60 years	169 (88%)	23 (12%)	
∆SPT ≥ 20°, *n* (%)	17 (10.1%)	3 (13.0%)	0.714
Impingement 40/20°, *n* (%)	28 (16.5%)	3 (13%)	>0.999
Patients >60 years	380 (70.6%)	158 (29.4%)	
∆SPT ≥ 20°, *n* (%)	57 (15%)	50 (31.6%)	**<0.001**
Impingement 40/20°, *n* (%)	104 (27.4%)	58 (36.7%)	**0.039**

*Note*: Bold values indicate statistically significant (*p* < 0.05).

Abbreviation: SPT, spinopelvic tilt.

Post hoc power analysis demonstrated that no calculation was possible in patients younger than 50 years, as no spinopelvic risk factors were present. In patients younger than 55 years, although theoretical power was very high (>99%), the results were not clinically interpretable given the very small number of patients with spinopelvic risk factors in this subgroup. In patients ≤60 years, the power was low (≈25%–28%) for detecting differences in ΔSPT ≥ 20° or impingement, which is consistent with the absence of significant associations in this group.

### ΔPFA and adverse spinopelvic mobility across age groups

**Figure 2:**
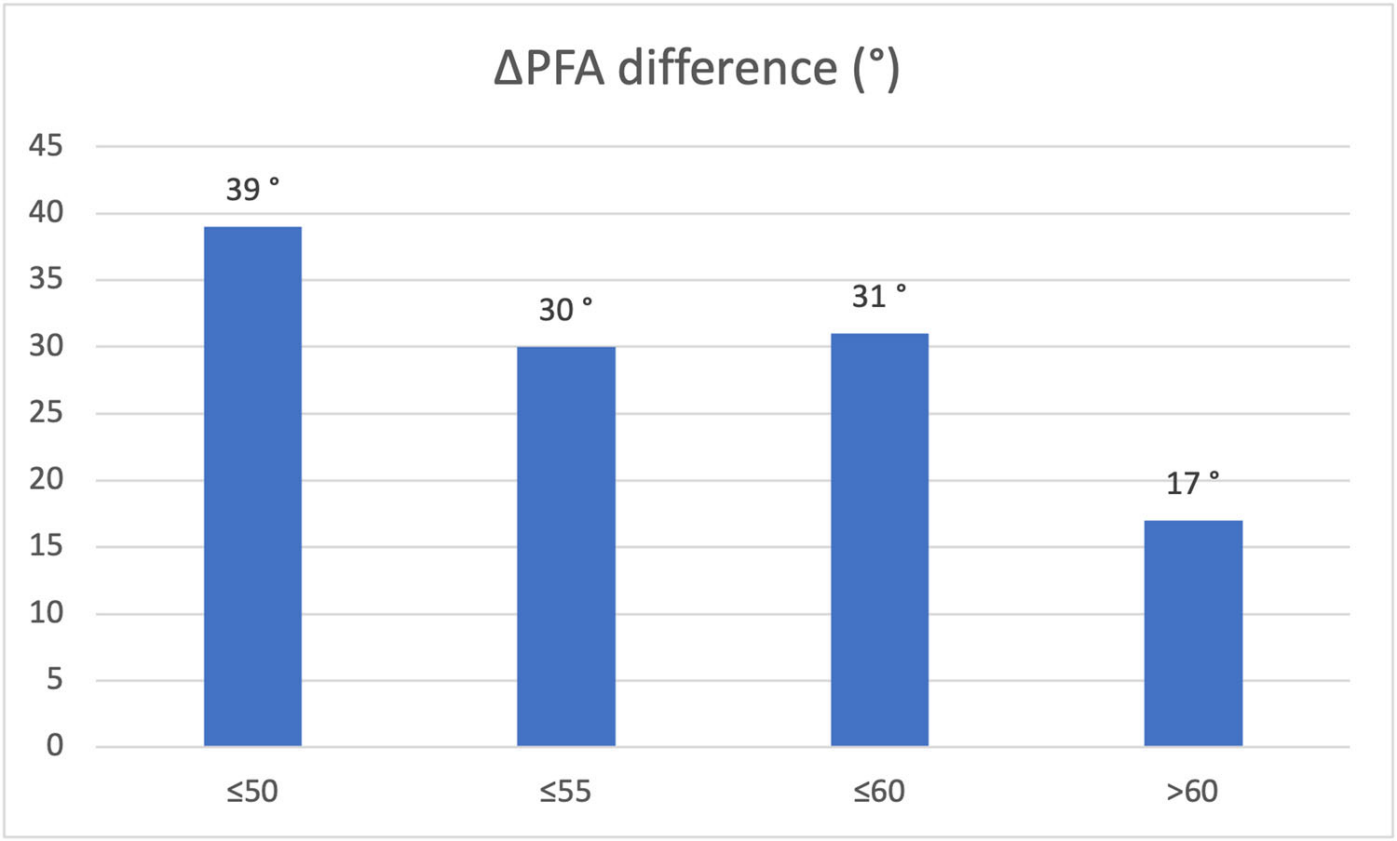
Differences in ΔPFA between patients with and without abnormal mobility (ΔSPT ≥ 20°) across age groups. Legend: Analysis restricted to patients without spinopelvic risk factors. The values represent the mean differences in the ΔPFA between patients with a ΔSPT < 20° and those with a ΔSPT ≥ 20° within each age group. PFA, pelvic femoral angle; SPT, spinopelvic tilt.

The ΔPFA increased progressively with age. The mean ∆PFA was significantly greater in patients with adverse spinopelvic mobility than in those without averse spinopelvic mobility across all age groups: **≤**50 years: 72.3° vs. 111.6° (+39°, *p* < 0.001), ≤55 years: 77.5° vs. 107.2° (+30°, *p* < 0.001), ≤60 years: 79.9° vs. 111.2° (+31°, *p* < 0.001) and >60 years: 91.0° vs. 108.0° (+17°, *p* = 0.01; Figure 2).

## DISCUSSION

Analysis of the hip–spine relationship is an important prerequisite for adapting implant positioning on the basis of spinopelvic mobility [29]. While several risk factors have been identified, they are related primarily to lumbar degeneration and tend to increase with age [20], which would theoretically place younger patients at low risk of adverse spinopelvic mobility.

The results of this study confirm that classical spinopelvic risk factors are uncommon in patients under 60 years of age and do not explain the occurrence of adverse spinopelvic mobility or the risk of impingement in this population, which is observed in approximately 10%–17% of patients under 60 years of age. In contrast, 29.4% of older patients presented with at least one spinopelvic risk factor, which was significantly associated with the risk of adverse spinopelvic mobility or impingement, with all comparisons reaching statistical significance. Although ΔPFA progressively increased with age, likely reflecting a compensatory mechanism for lumbar stiffness, the magnitude of the difference in ΔPFA between patients with and without adverse spinopelvic mobility was more pronounced in younger subgroups. In younger patients without spinopelvic risk factors, those with adverse spinopelvic mobility (ΔSPT ≥ 20°) displayed disproportionately high ΔPFA values, with mean differences of +30°–39° compared with patients with normal mobility. In contrast, in older patients, ΔPFA was globally elevated even in the absence of adverse spinopelvic mobility, and the difference between those with and without ΔSPT ≥ 20° was more modest (+17°). This pattern likely reflects an adaptive phenomenon to degenerative lumbar factors such as stiffness and sagittal imbalance [28].

Spinopelvic risk factors, including lumbar stiffness, sagittal imbalance, and posterior SPT in the standing position, play key roles in adverse spinopelvic mobility, increasing the risk of impingement and dislocation following THA [32, 33]. Prosthetic impingement is likely an underrecognized phenomenon, with in silico studies reporting rates as high as 30% with standard implant positioning [1, 8] and studies analysing revision THA cases reporting rates up to 50% [23]. In this study, not a single patient under 50 years of age met any of these criteria, raising questions about the clinical utility of spinopelvic risk factor screening in younger THA candidates.

**Figure 3:**
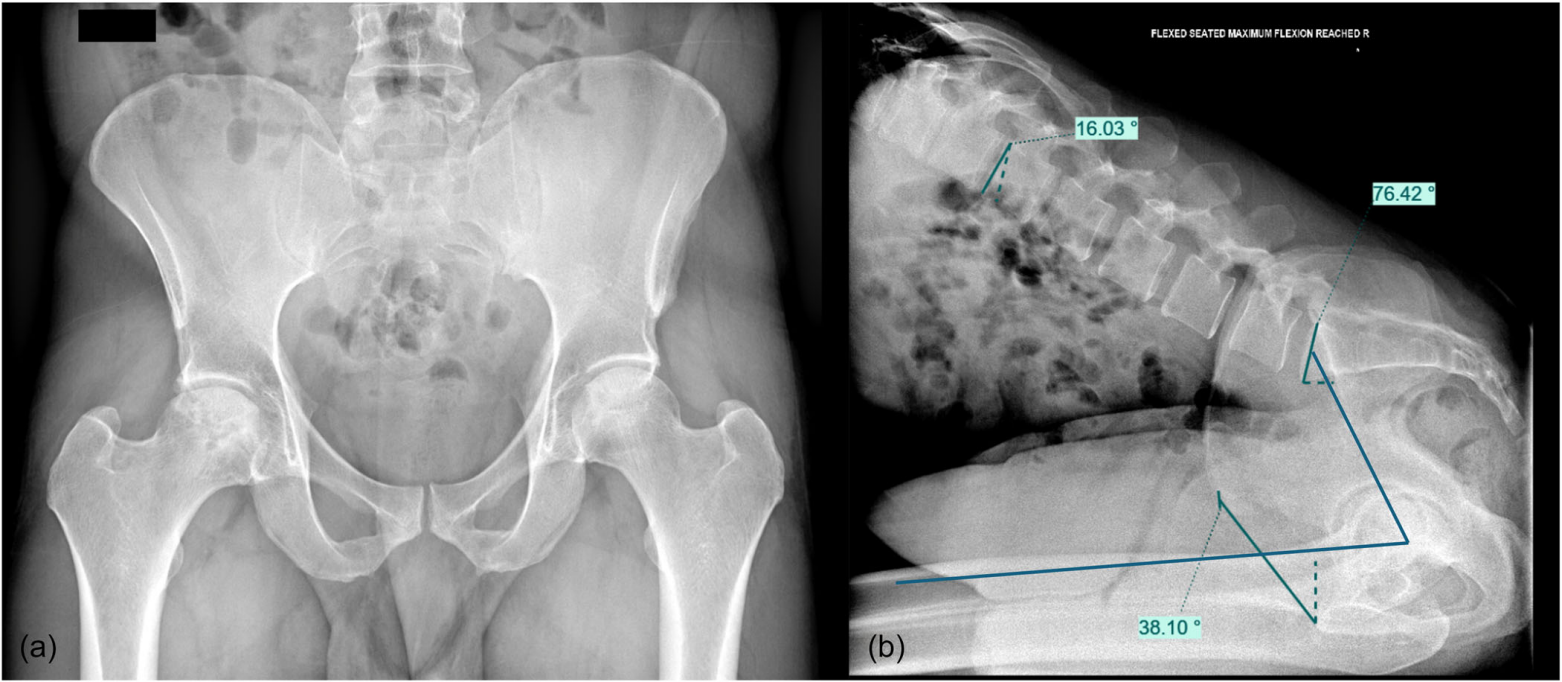
A 36‐year‐old woman with right femoral head osteonecrosis prior to THA. (a) Anteroposterior radiograph of the pelvis. (b) Lateral radiograph in the flexed‐seated position. Pelvic parameter analysis revealed no spinopelvic risk factors (spinopelvic tilt: 12°, lumbar flexion: 52°, PI: 52°, PI‐LL: −16°), but adverse spinopelvic mobility (26°) was associated with high hip mobility (∆PFA = 115°). LL, lumbar lordosis; PFA, pelvic femoral angle; PI, pelvic incidence; THA, total hip arthroplasty.

In the groups of younger patients, no significant differences in terms of risk factors were found between those with and without adverse spinopelvic mobility (∆SPT ≥  20°). The only parameter that significantly differed was hip mobility, reflected by a markedly greater variation in the pelvic femoral angle (∆PFA) in the adverse mobility group from the standing to the flexed seated position (Figure 3). These findings suggest that in younger patients, in whom lumbar and pelvic stiffness is uncommon, hip motion may contribute more directly to adverse spinopelvic kinematics. Recent studies support the notion that hip mobility plays a central role in functional alignment and stability after THA. Dorr et al. proposed that femoral hypermobility, defined as a sitting PFA < 115° or a relaxed‐seated ∆PFA > 75°–95°, is an independent risk factor for anterior impingement and posterior dislocation, particularly in high‐demand flexion postures such as deep seated positions [19]. Similarly, a study demonstrated that a ∆PFA ≥ 95° was necessary for observing adverse spinopelvic mobility ≥20° [5], further highlighting the role of the hip in driving pelvic compensation. This finding indicates that in younger patients, adverse spinopelvic mobility or the risk of impingement does not appear to be related to the classical degenerative spinopelvic risk factors usually observed in older populations. Instead, it seems to be driven by other mechanisms, primarily the greater hip flexion observed in this group. In addition, other anatomical parameters are likely to play a role, particularly femoral version and, more broadly, combined version, which have been shown to critically influence impingement risk. Taken together, these results support the value of a functional combined anteversion assessment [25] in younger patients, even in the absence of classical spinopelvic risk factors, and highlight the potential role of 3D and functional planning for optimising implant positioning [10].

The present study has certain limitations. First, the retrospective design may introduce selection bias and limit causal interpretation. Nevertheless, all patients were consecutively included from a prospective institutional registry with standardised imaging and assessment protocols, which reduces the risk of information bias and ensures data consistency.

Moreover, the number of patients under 55 years of age, especially those with adverse spinopelvic mobility, was relatively small, which resulted in low statistical power in the subgroup analyses. However, this reflects the true epidemiology of THA in younger populations, where risk factors are less common.

Third, we did not analyse the risk of dislocation in this retrospective study and analysed only the rates of adverse spinopelvic mobility and impingement. Instability and impingement are associated with other factors, including implant position, combined anteversion, leg length, offset and soft‐tissue tension [14, 17, 18], and should be predicted before surgery. The purpose of this study was only to understand the necessity of assessing spinopelvic risk factors and adverse spinopelvic mobility in young patients before THA.

For the impingement risk simulations, we selected a cup anteversion of 20°. Although some preoperative spinopelvic classifications suggest that higher anteversion may be preferable [30], an in silico analysis comparing different systematic orientations indicated that this value was associated with a lower impingement risk [8]. In addition, the simulations were performed using the native femoral stem version, but the final prosthetic femoral version may deviate from the native femoral version [9]. This limitation should be considered during THA planning.

Hip flexion (∆PFA) was analysed as a continuous parameter, and although its association with outcomes was robust, the absence of a defined threshold from the standing position to the flexed‐seated position prevents immediate clinical application. Prospective studies are warranted to validate ΔPFA as a functional marker and determine clinically relevant cutoff values.

Finally, while spinopelvic risk factors tend to remain relatively stable during the postoperative period, pelvic mobility may change significantly, driven primarily by the restoration of hip flexion [3, 12]. It is therefore possible that the rate of adverse spinopelvic mobility is underestimated preoperatively and may increase after the recovery of hip flexion in patients with osteoarthritis.

## CONCLUSIONS

Traditional risk factor‐based screening may underestimate the presence of adverse spinopelvic effects or the risk of impingement in younger THA candidates. In this population, where lumbar stiffness is uncommon, excessive hip motion may be the primary driver of adverse spinopelvic mobility. Therefore, preoperative evaluation should not rely solely on established risk criteria and should also include functional assessment to optimise implant orientation to potentially decrease the risks of impingement and dislocation.

## AUTHOR CONTRIBUTIONS

Thomas Aubert designed the study. Maxime Rodilla and Thomas Aubert wrote the manuscript. Maxime Rodilla, Thomas Aubert and Luc Lhotellier interpreted the data. Thomas Aubert performed the analyses. Maxime Rodilla and Luc Lhotellier participated in data interpretation and critically reviewed the manuscript. All authors revised the manuscript for important intellectual content.

## CONFLICT OF INTEREST STATEMENT

Thomas Aubert is a consultant for Corin and Depuy.

## ETHICS STATEMENT

IRB 01 2025_16Orth Croix Saint Simon.

## Data Availability

The data that support the findings of this study are available from the corresponding author upon reasonable request.
